# BdGUCD1 and Cyclic GMP Are Required for Responses of *Brachypodium distachyon* to *Fusarium pseudograminearum* in the Mechanism Involving Jasmonate

**DOI:** 10.3390/ijms23052674

**Published:** 2022-02-28

**Authors:** Maria Duszyn, Brygida Świeżawska-Boniecka, Monika Skorupa, Krzysztof Jaworski, Adriana Szmidt-Jaworska

**Affiliations:** 1 Department of Plant Physiology and Biotechnology, Faculty of Biological and Veterinary Sciences, Nicolaus Copernicus University in Toruń, Lwowska St. 1, 87-100 Torun, Poland; swiezawska@umk.pl (B.Ś.-B.); monika_skorupa@umk.pl (M.S.); jaworski@umk.pl (K.J.); asjawors@umk.pl (A.S.-J.); 2 Centre for Modern Interdisciplinary Technologies, Nicolaus Copernicus University in Toruń, Wilenska St. 4, 87-100 Torun, Poland

**Keywords:** guanylyl cyclase, 3′,5′-cyclic guanosine monophosphate, cGMP, PepR2, *Brachypodium distachyon*, *Fusarium pseudograminearum*, biotic stress, phytohormones, jasmonate

## Abstract

Guanosine 3′,5′-cyclic monophosphate (cGMP) is an important signaling molecule in plants. cGMP and guanylyl cyclases (GCs), enzymes that catalyze the synthesis of cGMP from GTP, are involved in several physiological processes and responses to environmental factors, including pathogen infections. Using in vitro analysis, we demonstrated that recombinant BdGUCD1 is a protein with high guanylyl cyclase activity and lower adenylyl cyclase activity. In *Brachypodium distachyon*, infection by *Fusarium pseudograminearum* leads to changes in *BdGUCD1* mRNA levels, as well as differences in endogenous cGMP levels. These observed changes may be related to alarm reactions induced by pathogen infection. As fluctuations in stress phytohormones after infection have been previously described, we performed experiments to determine the relationship between cyclic nucleotides and phytohormones. The results revealed that inhibition of cellular cGMP changes disrupts stress phytohormone content and responses to pathogen. The observations made here allow us to conclude that cGMP is an important element involved in the processes triggered as a result of infection and changes in its levels affect jasmonic acid. Therefore, stimuli-induced transient elevation of cGMP in plants may play beneficial roles in priming an optimized response, likely by triggering the mechanisms of feedback control.

## 1. Introduction

Cyclic nucleotides (cNMPs), adenosine-3′,5′-cyclic monophosphate (cAMP), and guanosine-3′,5′-cyclic monophosphate (cGMP), are involved in signal transduction in all living cells [1,2]. New information regarding the characterization of new enzymes, adenylyl and guanylyl cyclases (ACs; GCs), synthesizing cNMPs and the signal transduction pathways in which they can participate is continuing to increase [3,4,5,6,7,8]. The role played by cNMPs in plants is currently poorly understood. To date, cGMP and GCs are known to be involved in several physiological processes and responses to environmental factors, including stressors. In recent years, examples of cyclic nucleotide and phytohormone interactions have been observed, e.g., cGMP is involved in plant hormone signaling and alters phosphorylation of *Arabidopsis thaliana* root proteins [9], several hormones fluctuate in response to changes in cGMP concentration in tomato [10], cGMP interacts with ABA-induced changes in H_2_O_2_ and NO and stimulates increased concentration of free Ca^2+^, leading to changes in the signaling pathway that causes stomatal closure in *A. thaliana* [11], *Oryza sativa* cGMP-dependent protein kinase mediates NO-cGMP signaling in response to GA [12], cGMP promotes ethylene production, and enhances the perception of ethylene [13]. While all of these findings collectively support diverse roles of cyclic nucleotides in plant development and environmental responses, the underlying molecular mechanisms are not well understood. Moreover, there is still much missing information and gaps in the understanding of the interactions between cGMP and hormones in response to pathogen infection. There is evidence that cGMP, GCs [4,5,14], and hormones are involved in the plant response to pathogen infection [15,16,17,18]. Therefore, it is worth exploring whether and how these molecules cooperate.

Fusarium crown rot (FCR) caused by the fungal pathogen *Fusarium pseudograminearum* is a disease that results in major yield and quality losses in many economically important plant species worldwide, including cereals. Although *Fusarium* utilizes multiple infection strategies, these fungi are considered to be hemibiotrophs capable of transitioning to necrotrophs depending on the specific environmental and metabolic stimuli. More specifically, infected seedlings can die before or after emergence, and typical disease symptoms of surviving seedlings include browning of the coleoptile, subcrown internode, lower leaf sheaths, and adjacent stems and nodal tissues. Browning can become evident within a few weeks after planting or at any time during plant development. Moreover, infected plants may develop white heads without or with shriveled grains [19].

The aim of our research was to determine the enzymatic activity of the new, putative guanylyl cyclase GUCD1 from *Brachypodium distachyon* and to analyze its involvement in changes in the level of cyclic nucleotides as a manifestation of the response to infection. Our preliminary studies have shown that cyclic nucleotide levels are altered following *B. distachyon* infection by *F. pseudograminarum*. This prompted us to determine the signaling elements involved in the inoculation and infection process in the above experimental setup. This study provides novel insights into the roles of cGMP signaling elements in cereal reactions to fungal pathogens. Furthermore, our results provide a basic framework for further research on cGMP signaling in biotic stress responses.

## 2. Results and Discussion

Much attention is currently focused on unraveling the molecular mechanisms involved in plant signal transduction cascades in the infection process. The steps between pathogen perception and the initiation of cellular defense response programs in plants remain only partially understood. The presented work supplements this knowledge with respect to cyclic nucleotides and purine nucleotide cyclases.

### 2.1. Sequence Analysis of BdGUCD1

The amino acid sequence of BdGUCD1 is referred to in the NCBI database as a Guanylyl Cyclase Domain Containing 1 (NCBI: XP_003568333.1). Analysis of predicted 268-aa amino acid sequences using the BlastP program revealed similarities in the BdGUCD1 protein compared to other monocots of putative guanylyl cyclases, with the highest identity score being with *H. vulgare* (XP_044978661.1; 90% identity), *P. miliaceum* (RLN00560.1; 85%), and *T. dicoccoides* (XP_037471862.1; 90%). In addition, the sequence shared homology with previously characterized GCs, such as ZmGC1 (ABD18446.1; 83%) [20], HpGC1 (ADJ94125.1; 64%) [5], AtGC1 (AAM51559.1; 55%) [21], and PnGC1 (ABG67691.2; 52%) [22].

There are two groups of GCs. The first, with canonical GC domains, often appears as a stand-alone protein. In the second, the cyclase domain is part of a larger, multidomain protein complex. This group includes so-called moonlight proteins, which in addition to the GC domain also have kinase domains. The GC domains of these proteins contain 14-amino acid active centers [21,23,24]. Determination of conserved residues within these predicted 14-amino acid catalytic centers led to the discovery of a number of candidate molecules with guanylyl cyclase activity. However, as mentioned earlier in the BdGUCD1 sequence, such motif was not found. It seems that such situation is no exception as neither the PnGC1 [22] nor HpGC1 [5] have such a motive. The lack of a motive while maintaining cyclase activity is very important information. The discovery may indicate that other cyclases may exist in plant cells, without the previously sought 14-amino acid catalytic motif or with other domains responsible for cyclase activity.

### 2.2. In Vitro Analysis of the AC and GC Activity of BdGUCD1

**Figure 1:**
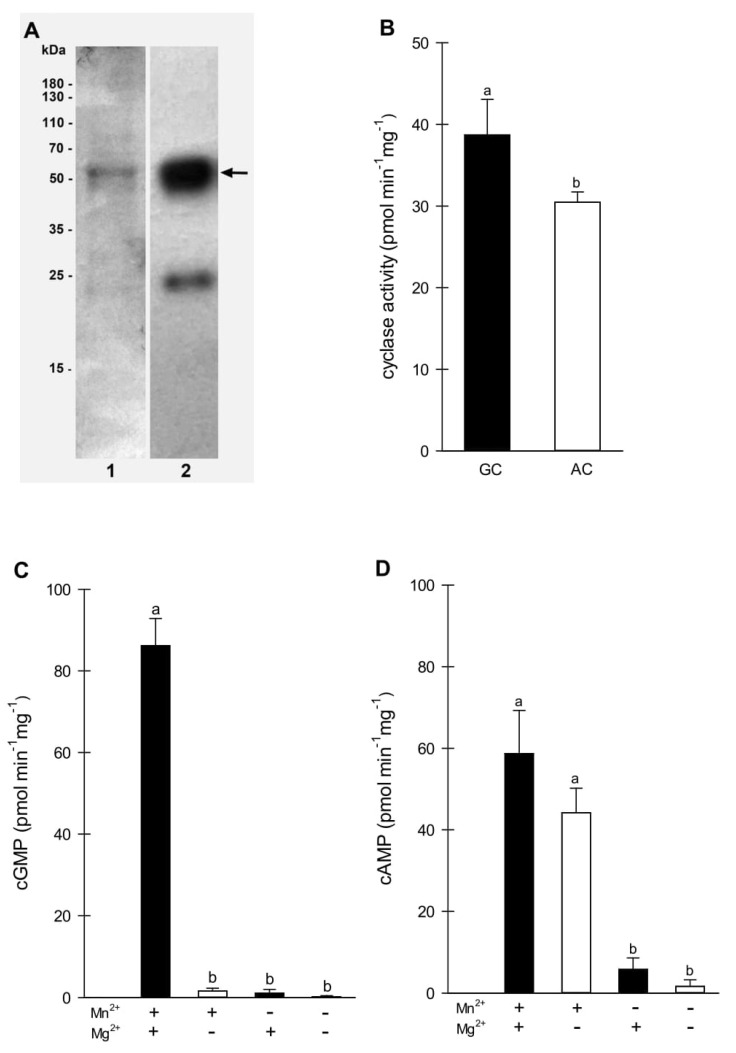
Enzymatic activity of recombinant BdGUCD1. (**A**) SDS–PAGE (1) and Western blot analysis using anti-GST antibodies (2) of BdGUCD1. The arrow indicates the position of the analyzed protein; (**B**) determination of BdGUCD1 substrate specificity. The reaction mixture contained 50 mM Tris/HCl buffer (pH 7.5), 5 mM MnCl_2_, 5 mM MgCl_2_, 1 mM GTP, 1 mM ATP, and 5 μg of the purified protein in a final volume of 100 μL; (**C**) determination of BdGUCD1 cofactor specificity for guanylyl cyclase activity. The reaction mixture contained 50 mM Tris/HCl buffer (pH 7.5), 5 mM MnCl_2_ and/or 5 mM MgCl_2_, 1 mM GTP, and 5 μg of purified protein in a final volume of 100 μL; (**D**) determination of BdGUCD1 cofactor specificity for adenylyl cyclase activity. The reaction mixture contained 50 mM Tris/HCl buffer (pH 7.5), 5 mM MnCl_2_ and/or 5 mM MgCl_2_, 1 mM ATP, and 5 μg of purified protein in a final volume of 100 μL. Data are shown as mean values (*n* = 3), and error bars indicate the standard error of the mean. Different letters above the bars indicate significant differences at *p* < 0.05.

The truncated 624-bp fragment of the *BdGUCD1* cDNA was cloned into the pGEX-6P-2 vector in frame with a glutathione S-transferase (GST) tag and expressed in *E. coli* BL21 cells as a GST-BdGUCD1 recombinant protein. The molecular mass of the 207-aa-long BdGUCD1 polypeptide was predicted in silico to be 29.78 kDa (while GST-BdGUCD1 is ~55 kDa), and the isoelectric point was predicted to be 4.97 (http://web.expasy.org/compute_pi; accessed on 17 December 2021). The recombinant BdGUCD1 protein was purified and used for GC enzymatic activity determination. Affinity chromatography enabled purification of the GST-BdGUCD1 fusion protein as a main 55 kDa band (Figure 1A). The preliminary experiments and obtained results showed that the foreignness of GST did not affect the enzymatic activity of the proteins tested.

The purified intracellular domain of the BdGUCD1 protein was tested for its ability to convert GTP substrate to cGMP product (or ATP to cAMP) in the presence of magnesium and/or manganese ions as cofactors. For GC activity, the maximal BdGUCD1 activity was reached at 1 mM GTP 15 min after starting the reaction and the generated level of cGMP was 86.17 pmol mg protein^−1^ min^−1^ (±5.44) (Figure 1C). However, adenylyl cyclase activity was lower and reached 58.70 pmol cAMP mg protein^−1^ min^−1^ (±8.63) (Figure 1D). The values shown above apply to variants where one of the nucleotides (only GTP or only ATP) was present in the reaction mixture. However, lower AC activity was observed when both GTP and ATP were added to the reaction mix (Figure 1B). This indicates that BdGUCD1 is a protein with dual activity, but its substrate specificity for GTP is stronger. The kinetic parameters determined for GC activity of BdGUCD1 were a V_max_ of 149.9 fmol min^−1^ ug^−1^ protein and a K_m_ value of 0.73 mM and for AC activity V_max_ 76.74 fmol min^−1^ ug^−1^ protein and a K_m_ 0.32 mM. The BdGUCD1 GC activity was similar to the activity described for PnGC1 (78.1 pmol mg^−1^ min^−1^) [22], BdPepR2 (72.1 pmol mg^−1^ min^−1^) [25], and BdERL1 (70 pmol mg^−1^ min^−1^) [26], but was higher compared to most other plant guanylyl cyclases. The different requirements for cofactors in the cyclase activity of BdGUCD1 are worth noting. Despite the lack of the 14-amino-acid catalytic motif characteristic of GC with the described amino acid responsible for magnesium or manganese binding, BdGUCD1 requires the presence of these ions for its activity. In the case of GC activity, both Mn^2+^ and Mg^2+^ ions are necessary (Figure 1C). However, for AC activity, only Mn^2+^ is needed (Figure 1D). This may be related to the regulation of BdGUCD1 activity depending on the ions present in the environment.

As mentioned previously, the 14-amino-acid catalytic motif was not found in the BdGUCD1 sequence. However, we have confirmed the high guanylyl and lower adenylyl activity. This may indicate the existence of a cyclase group with a different structure and catalytic domain. It is worth mentioning here that proteins with confirmed GC activity often have additional phosphodiesterase, AC, or kinase activity modulated by cyclic nucleotides or calcium, which presumably may affect GC activity and may be important for the various roles of these enzymes in plant cells.

### 2.3. Effect of F. pseudograminearum Treatment on BdGUCD1 and BdPepR2 Expression and Endogenous Levels of Cyclic Nucleotides

The available data in the literature indicate that cGMP and enzymes with in vitro confirmed GC activity are involved in the response to pathogen infection [4,5,6,7,27,28]. Active resistance is triggered by pathogens and involves biochemical and molecular responses. Pathogens produce elicitors called pathogen/microbe-associated molecular patterns (PAMPs/MAMPs), including peptides, metabolites, cell wall components, enzymes, and toxins, to suppress plant defense. Following pathogen attack, the damaged host produces damage-associated molecular patterns (DAMPs), including plant signal molecules. These elicitors or PAMPs/MAMPs/DAMPs are recognized by pattern recognition receptors (PRRs). Among the “moonlighting kinases with GC activity”, some proteins act as receptors that recognize PAMPs/DAMPs and activate defense against pathogens. PRRs initiate downstream signaling, and then PRR-derived signals are transmitted via further phosphorylation cascades [29]. Among the *A. thaliana* DAMPs, there is a family of AtPep1–6 peptides. Most likely, each AtPep binds to the plant cell plasma membrane AtPepR1 receptor, which also belongs to an LRR-RLK with GC activity. It was speculated that the binding of AtPep to the AtPepR1 receptor causes local elevation of cGMP, which subsequently leads to the activation of a plasma membrane-localized cyclic nucleotide-gated, Ca^2+^-conducting channel and CNGC-dependent cytosolic Ca^2+^ elevation [6].

**Figure 2:**
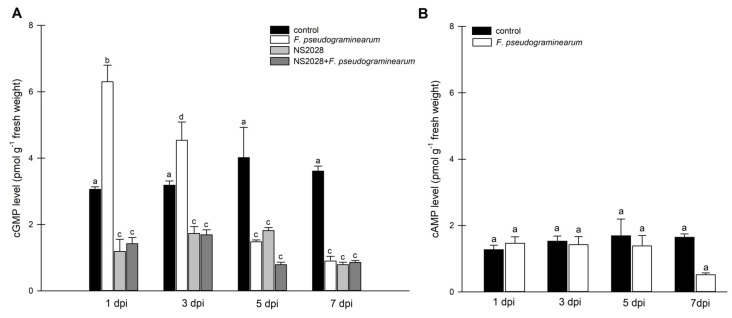
Determination of intracellular cyclic nucleotides levels in *B. distachyon* plants infected with *F. pseudograminearum.* (**A**) Time course of cGMP generation in B. distachyon in response to F. pseudograminearum infection and inhibitor (NS 2028) treatment; (**B**) time course of cAMP generation in *B. distachyon* in response to *F. pseudograminearum* infection. Control plants were not infected or treated with the inhibitor. The data are shown as mean values (*n* = 3), and error bars represent the standard error of the mean. Different letters above the bars indicate significant differences at *p* < 0.05 (ANOVA followed by Tukey’s test).

To supplement the existing state of knowledge, we attempted to determine the role of *BdGUCD1*, *BdPepR2* [25], and cyclic nucleotides during the stress response. The endogenous levels of cGMP and cAMP and the quantitative expression levels of the *BdGUCD1* and *BdPepR2* genes were measured at four time points after *F. pseudograminearum* infection (1, 3, 5, and 7 dpi). In control 3-week-old *B. distachyon* plants, endogenous cGMP levels oscillate at approximately 3 pmol g^−1^ fresh weight and double 24 hours after infection. Three days after infection, levels consecutively decreased, but remained statistically higher than that in the control variant and then decreased in subsequent days (Figure 2A). In contrast, the levels of cAMP remained unchanged after *F. pseudograminearum* infection compared to control variants throughout the entire 7-day experiment (Figure 2B). These data confirmed that only cGMP is involved in the plant’s response to infection in agreement with previous observations when pathogen infection causes the accumulation of cGMP in *A. thaliana* plants and cGMP played a key role in local responses controlling the induction of systemic acquired resistance [30]. Furthermore, the accumulation of cGMP in *A. thaliana* was observed hours after contact with avirulent *Pseudomonas syringae* strains. Moreover, this observation can be linked to the immediate recognition of specific pathogen avirulent gene-encoded molecules by resistance genes. This early detection of the pathogen and activation of the defense response involves the production of a range of signaling molecules, such as reactive oxygen species, NO, JA, SA, ethylene, and transcriptional activation of defense-related genes [28]. Changes in endogenous cGMP levels were also observed in *Hippeastrum hybridum* after mechanical wounding and fungal infection [5] and in *A. thaliana* seedlings subjected to salt stress [31].

**Figure 3:**
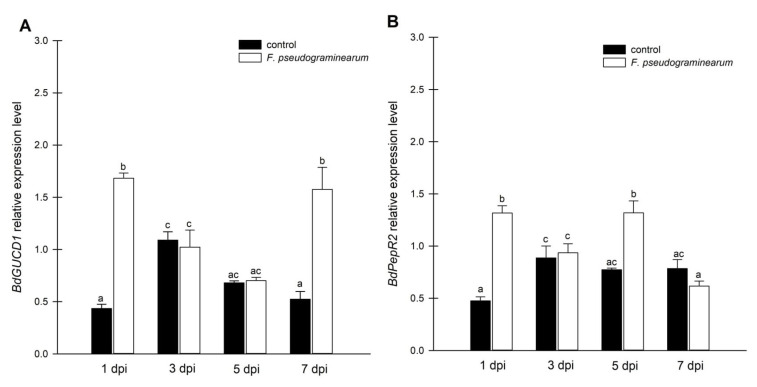
Expression analysis of guanylyl cyclase genes in *F. pseudograminearum*-infected *B. distachyon* plants. (**A**) mRNA levels of *BdGUCD1* in *B. distachyon* in response to *F. pseudograminearum* infection; (**B**) mRNA levels of BdPepR2 in *B. distachyon* in response to *F. pseudograminearum* infection. Control plants were not infected or treated with the inhibitor. Different letters above the bars indicate significant differences at *p* < 0.05 (ANOVA followed by Tukey’s test).

Cellular cNMP levels in plants represent the pooled outcome of cyclase (and PDE) activities expressed by different genes of diverse biological functions. To examine the observed changes in cGMP to enzyme activity, we focused on expression analysis of the *BdGUCD1* and *BdPepR2* genes, which encode proteins with guanylyl cyclase and kinase activity, and determining their expression profile in response to *F. pseudograminearum* infection. BdPepR2 is a plasma membrane receptor of peptide signalling molecules with confirmed guanylyl cyclase activity [25], while BdGUCD1 is presumed to be a cytosolic protein. The data showed that during the 7-day test cycle after fungal infection expression of both genes fluctuated. The mRNA levels of *BdGUCD1* were significantly upregulated the day after infection, then decreased and peaked 7 days after infection (Figure 3A). With respect to *BdPepR2*, the highest expression levels with regard to the control were observed 1 and 5 days after infection (Figure 3B). *F. pseudograminearum* caused changes in expression levels of both genes at the beginning (a rapid threefold increase was observed in both genes one day after infection) and later stages of the stress response (5 dpi in the case of *BdPepR2* and 7 dpi in *BdGUCG1*). These changes in mRNA levels are consistent with previous hypotheses, since the *BdPepR2* gene encodes a protein presumably acting as a receptor responsible for pathogen protein recognition. This significant induction of *BdPepR2* gene expression stimulated by *F. pseudograminearum* may suggest a role for the BdPepR2 protein in the activation of the plant defense response. Importantly, an increase in gene expression does not guarantee an increase in the corresponding protein abundance, because transcription is only one of the levels of regulation of protein expression. Of note, the profile of *BdPepR2* gene expression coincided with the peak in endogenous cGMP levels one day after *F. pseudograminearum* inoculation (Figure 2A). It has previously been shown that the accumulation of *HpPepR1* transcript was sharply increased after fungal infection, whereas mechanical wounding had no effect on the expression profile of the studied gene [4]. Together, these results may indicate participation of the cGMP-dependent pathway in alarm plant reactions induced by pathogen infection. We showed that BdPepR2 is proteins with guanylyl cyclase and kinase activity, which is modulated by cGMP [20]. So the dual activity of the BdPepR2 may explain the elevated transcript levels 5 days after infection when cGMP levels remain low. Among the group of “moonlighting kinases with GC activity”, two other proteins, AtWALK−10 and AtPepR1, act as receptors that recognize PAMPs/DAMPs and activate defense against pathogens. Expression of AtWAKL10 was shown to be induced by both biotrophic and necrotrophic pathogens, or their elicitors, and it was suggested that this indicates the participation of AtWAKL10 in the response to a broad range of pathogens [28]. Among *A. thaliana* DAMPs is a family of AtPep1–6 peptides. Most likely, each AtPep binds to the plant cell plasma membrane AtPepR1 receptor, which also belongs to an LRR-RLK with GC activity. The authors suggested that the binding of AtPep to the AtPepR1 receptor induces local elevation of cGMP, subsequently leading to the activation of a plasma membrane-localized cyclic nucleotide-gated, Ca^2+^-conducting channel and CNGC-dependent cytosolic Ca^2+^ elevation [6]. Moreover, two tomato homologues of *A. thaliana* PEPRs, SlGC17 and SlGC18, exhibited in vitro GC activity. Cosilencing of *SlGC17* and *SlGC18* genes significantly attenuated resistance to tobacco pathogens and reduced PAMP- and DAMP-triggered immunity by decreasing flg22-, chitin-, and AtPep1-elicited Ca^2+^ and H_2_O_2_ bursts [27]_._ These results indicate how important the role of moonlighting proteins and proteins with GC activity can be in plant responses to biotic stressors. This significant induction of both genes, *BdPepR2* and *BdGUCD1*, observed 1 day after infection may be an indication of the importance of cGMP in response to fungal infection. 

### 2.4. Effect of F. pseudograminearum Treatment on Phytohormone Levels

Both salicylic acid (SA) and jasmonic acid (JA), so-called immunity hormones, are involved in stress reactions and basal and/or induced resistance against plant pathogens. Importantly, they do not act independently from each other, but rather form a network of interactions in response to pathogens with different lifestyles. Once the SA pathway is activated at the site of infection, a defense response is triggered in distal plant parts to protect undamaged tissues. Mutants insensitive to SA or defective in SA accumulation display enhanced susceptibility to pathogens. Moreover, an increase in SA levels in pathogen-exposed tissues leads to the induction of pathogenesis related (PR) genes [32]. Many of the *F. pseudograminearum*-responsive genes are altered by the toxin and by plant defense-related hormones, such as SA and JA [19]. In *A. thaliana,* SA and JA interact antagonistically, which can modulate defense gene expression in response to pathogen infection [33]. JA and SA signaling were found to co-induce broad-spectrum disease-response genes, co-repress genes related to photosynthesis, auxin, and gibberellin, and reallocate resources of growth towards defense [34]. However, the detailed relationships between these hormones and other molecules are still unknown and worth further investigation.

**Figure 4:**
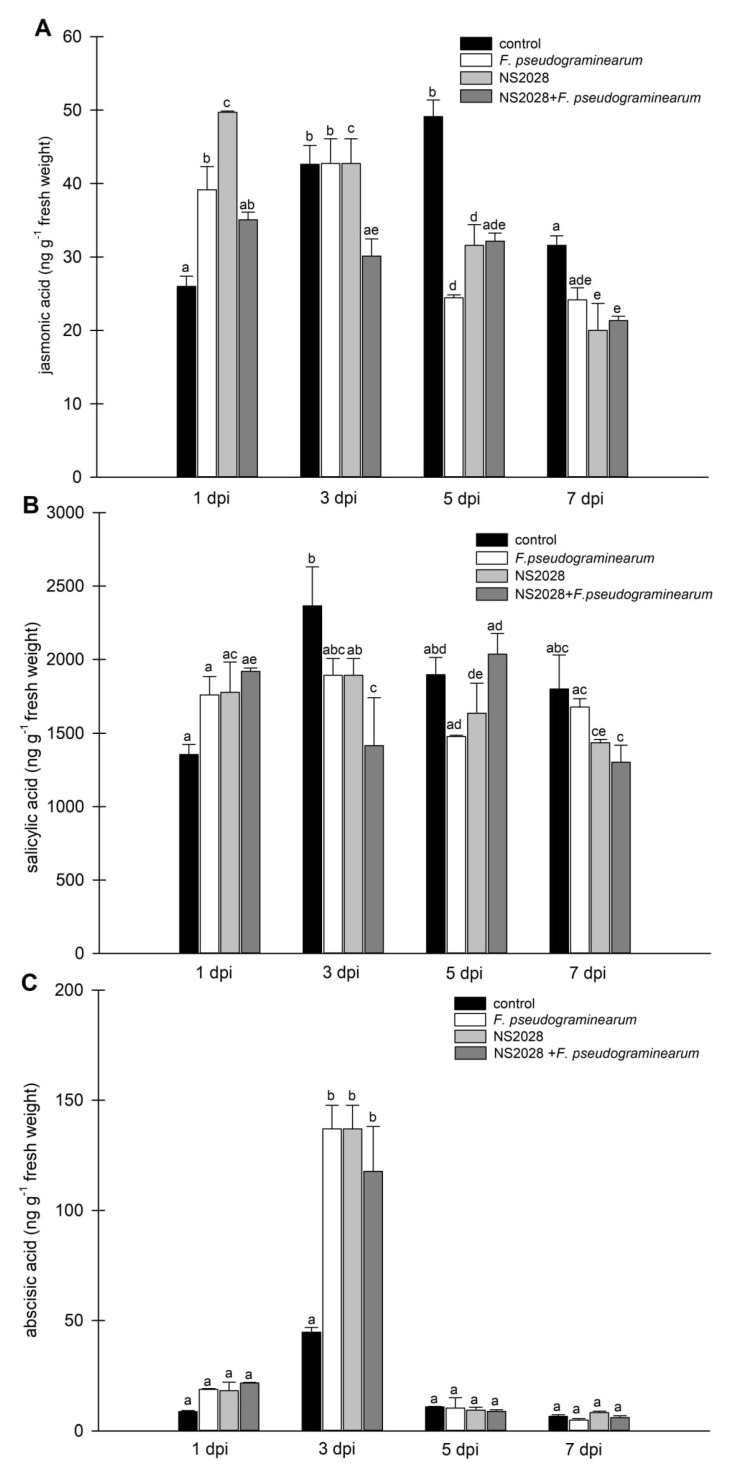
Determination of endogenous levels of phytohormones in *B. distachyon* infected with *F. pseudograminearum*. (**A**) Jasmonic acid; (**B**) salicylic acid; and (**C**) abscisic acid. Control plants were not infected or treated with the inhibitor. Data are shown as mean values (*n* = 3), and error bars indicate the standard error of the mean. Different letters above the bars indicate significant differences at *p* < 0.05 (ANOVA followed by Tukey’s test).

To determine whether infection with the necrotrophic fungal pathogen *F. pseudograminearum* alters phytohormone levels in *B. distachyon*, we quantified SA, JA, and ABA levels using LC–MS/MS in a time course experiment. The results showed that JA levels increased at 1 dpi, but not at later time points. Additionally, SA levels rose at 1 dpi and a significant decrease compared to the control was noted (Figure 4). These results are consistent with a previous report, implying that JA acts as a primary responsive signal molecule during *F. pseudograminearum* infection in monocots [35,36]. Three days post germination, *B. distachyon* seedlings were treated with *F. pseudograminearum* induction of genes encoding plant hormones, and three defense-related phytohormones (JA, SA, and ABA) were observed at 3 and 7 dpi [37]. In the case of wheat, only SA levels were increased at 1 dpi, while JA levels were unchanged [38]. This confirms involvement of these phytohormones in the plant response to stress at various stages of development in monocots. However, all data concerning stress hormones were inconsistent and demonstrated induction of different hormones at different times in different plants. Changes in hormone levels can also be related to the stress response to cell damage during the first stages of responses. However, it is not possible to separate these stages, so it should be considered a larger process involving the response of plants to wounding and pathogen infection.

ABA appears to be a key regulator of defense against necrotrophs with both negative and positive contributions. Clarification of the nature of ABA function is further confounded by its interactions with other resistance pathways and the potential trade-offs resulting from the occurrence of abiotic stresses during infection [39]. Current knowledge indicates that ABA influences some responses of plant pathogen resistance (e.g., stomatal closure and red-ox homeostasis), although its effect may vary with a number of variables, such as type of tissue, age of the plant, pathogen type, and stage of the infection [40]. In our experiments, a significant change in ABA levels was observed up to three days after infection. ABA levels tripled on day three. Together with the infection progress (5–7 dpi), a dramatic drop in ABA concentration was noted. A similar relationship was recently observed in flax [41]. Upregulation of the terpenoid pathway and increased ABA content in flax upon *Fusarium* infection leads to activation of the early plant response (PR genes, cell wall remodeling, and redox status). In this system, levels of accumulated ABA strongly increased continuously during the first 36 h. Transgenic flax plants with an elevated ABA level showed increased resistance to fusariosis [41], which may be associated with higher expression of the chitinase gene. This was also shown in wheat, where in a more resistant cultivar, twofold higher levels of ABA were noted [42]. Therefore, we conclude that the elevated synthesis of ABA correlates with *B. distachyon* responses to *F. pseudograminarum* and is involved in the early response of the plant to infection. Isner et al. investigated potential interactions between phytohormones and cGMP signaling and showed that plant hormones (ABA, IAA, JA) evoke rapid and concentration-dependent changes in cellular cGMP. However, brassinosteroids and cytokinin did not affect the levels of cGMP [9]. There is some evidence of a direct connection or the lack of a direct connection between ABA and cGMP, so it appears to be whole plant, organ, or process dependent [11,43,44,45,46]. However, it can also be associated with the use of different methods to study the changes in the endogenous level of these molecules, which has become more accurate over time.

### 2.5. Impact of cGMP on Phytohormone Levels during Infection

The results presented above indicate the involvement of cyclic GMP in the response of *B. distachyon* to stress induced by *F. pseudograminearum* infection. Therefore, we also wanted to investigate whether any interactions between cGMP and hormones is observed during the response to stress. We administered the guanylate cyclase inhibitor (NS 2028) the day before infection and measured the levels of all molecules tested at the same time as previous variants. NS 2028 was previously used in the characterization of PnGC-1 [22] and plant natriuretic peptides [44]. Most importantly, endogenous levels of cGMP in the variants treated with NS 2028 were significantly lower than that in the control plants (Figure 2A). This confirms that NS 2028 blocked the activity of plant guanylate cyclases, resulting in low levels of cGMP. The inhibitor was not affected by the infection, guanylyl cyclase was still inhibited, and cGMP levels remained low.

Application of the inhibitor caused increased levels of JA compared to the control at the first analyzed time (1 dpi); at subsequent times, the values were similar or lower (Figure 4A). In the case of NS 2028, application of SA and ABA maintained a similar pattern, so the levels of these hormones changed similarly from day to day after administration of the inhibitor (and *F. pseudograminearum*) compared to the results discussed above (Figure 4B,C). In line with these previous findings, we showed that cellular cGMP reduction modulated the levels of jasmonic acid in plants. Based on the available data, it can be concluded that this is an indirect effect. Inhibition of cGMP levels may strengthen JA signal transduction-mediated production of defense elements, but this needs to be validated by further experiments.

In conclusion, these advances are very limited and represent the beginning of elucidating cyclic nucleotide signaling cascades in combination with hormones that require comprehensive and systemic research. The role for cGMP signaling in the action of phytohormones has previously been shown both in growth and development, as well as in responses to stress factors [47]. A strong link between brassinosteroids and cGMP has been described [48]. GMP has also been linked to auxins in the context of root development, primarily due to its link with NO [49]. Studies on barley germination inferred a strong link between gibberellin (GA) and cGMP [43]. Isner et al. [9] showed that ABA induced cGMP in rice protoplasts seconds after treatment. Salt and osmotic stress are known to increase ABA levels and induce a rapid increase in cGMP levels in Arabidopsis seedlings [31]. cAMP was suggested to act upstream of salicylic acid (SA) during the plant defense process and induce PR1 expression [50] and it modulates the jasmonic acid (JA)-mediated signaling pathway [51]. The basis of the changes observed in these studies is not known, further complicated by the existence of a dependency network. While there are dependencies between these elements, we are only beginning to elucidate cyclic nucleotide signaling cascades in combination with hormones, which requires continued comprehensive and systemic research.

## 3. Materials and Methods

### 3.1. Construction of the Expression Vector, Expression and Purification of Recombinant Protein

Total RNA was extracted from 3-week-old *Brachypodium distachyon* Bd21 seedlings using a Universal RNA Purification Kit (EURx, Poland). cDNA was synthesized using the NG dART RT kit (EURx, Poland). A 624-bp fragment of the *BdGUCD1* (NCBI accession number: XM_003568285.3; https://www.ncbi.nlm.nih.gov/nuccore; accessed on 27 March 2018) coding region corresponding to a 207-residue polypeptide [Met^62^→Leu^268^] was amplified by PCR using specific primers (Table S1). Next, the PCR product was introduced into the linearized pGEX-6P-2 expression vector using In-Fusion cloning technology (In-Fusion HD Cloning Kit; Takara Bio USA, Inc., Mountain View, CA, USA). After being transformed with the resulting plasmid, the *E. coli* BL21 strain was used to produce the glutathione S-transferase (GST)-tagged protein. For the expression of BdGUCD1 recombinant protein, the construct was transformed into One Shot BL21 (DE3)pLysS *E. coli* cells (Life Technologies, Carlsbad, CA, USA). Bacterial cells were grown in LB medium supplemented with 2% glucose at 37 °C. Fusion protein production was induced by adding IPTG at a final concentration of 1 mM and incubating the cells at 22 °C for 3 h in glass vessels connected to a BioFlo 120 bioprocess control station (Eppendorf, Hauppauge, NY, USA). The pH was maintained at 6.5 (±0.2), the dissolved oxygen parameter was set to 20%, and the agitation speed was 200 rpm. Bacteria were collected by centrifugation and proteins were purified, as previously described [5]. The homogeneity and purity of the protein fractions were analyzed using 10% (*v*/*v*) SDS/PAGE and Western blotting analysis, as previously described [52].

### 3.2. Determination of Guanylyl and Adenylyl Cyclase Activity

Guanylyl and adenylyl cyclase activity was determined by estimating the rate of cGMP or cAMP formation. For the enzymatic assay, the reaction mixture contained 50 mM Tris/HCl buffer (pH 7.5), 5 mM MnCl_2_ or/and 5 mM MgCl_2_, GTP and/or ATP as a substrate (1 mM), and 5 μg of the purified protein in a final volume of 100 μL. After incubation at 30 °C for 15 min, the reaction was stopped at 100 °C for 10 min and the samples were centrifuged at 16,000× *g* for 10 min. Preliminary trials were also performed to determine the optimal reaction conditions (data not shown). Moreover, background cGMP levels were measured in tubes that contained the reaction mixture without protein. Total cGMP/cAMP concentration was determined in triplicate using liquid chromatography-tandem mass spectrometry (LC–MS/MS Nexera UHPLC and LCMS-8045 integrated system (Shimadzu Corporation, Kyoto, Japan)). The ionization source parameters were optimized in positive ESI mode using pure cGMP (or cAMP) dissolved in HPLC-grade water. Samples were separated using a reversed-phase C18 column (150 mm × 2.1 mm, 2.6 µm, Kinetex) in 10% methanol with 0.1% (*v*/*v*) formic acid (solvent A (water with 0.1% (*v*/*v*) formic acid), solvent B methanol with 0.1% (*v*/*v*) formic acid) at a flow rate of 0.3 mL/min. The interface voltage was set at 4.0 kV for positive (ES+) electrospray. Data acquisition and analysis were performed using the LabSolutions workstation for LCMS-8045. Enzyme activity was defined as the amount of cGMP or cAMP produced by 1 mg of protein per min.

### 3.3. Plant Material and Pathogen Inoculation

*Brachypodium distachyon* Bd21-1 was grown in a growth cabinet under long-day conditions (16/8 light/dark) at 24 °C. The *Fusarium pseudograminearum* isolate (laboratory code F0444) was obtained from GIORiN (Main Inspectorate of Plant Health and Seed Inspection; Toruń, Poland). Inoculum preparation was as described by [38]. Briefly, the inoculum was prepared using PDA plates inoculated for 10 days at 24 °C. Subsequently, spores were produced in mung bean broth by inoculation with agar plugs (0.5 mm) taken from the *F. pseudograminearum* plate culture and incubating on an orbital shaker at room temperature (15 rpm; ~22 °C) for 9 days. After filtration and centrifugation, spores were suspended in distilled water to a final concentration of 1 × 10^6^ spores ml^−1^ (spores concentration was measured by visual counting with a hemocytometer). Three-week-old *B. distachyon* plants were treated with an *F. pseudograminearum* suspension. Some variants of the experiment were treated with 1 mM NS 2028 24 h prior to infection with *F. pseudograminearum*. The solution was precisely applied using a tiny brush on the leaves. Plant material was collected 1, 3, 5, and 7 dpi (days post-infection), immersed in liquid nitrogen, and stored at −80 °C. Frozen plant material was manually homogenized using a pre-cooled mortar and pestle with liquid nitrogen and stored in -80 °C for subsequent experiments, which were performed in triplicate.

### 3.4. Determination of Endogenous Levels of Phytohormones

Endogenous levels of abscisic acid (ABA), jasmonic acid (JA), and salicylic acid (SA) were measured using mass spectrometry combined with liquid chromatography (LC–MS). Phytohormones were extracted using a modified QuEChERS-based method [53]. Extraction buffer (80% acetonitrile, 5% formic acid (FA), 15% water, and 1 mM butylhydroxytoluol (BHT)) was added to a sample containing 100 mg of leaves homogenized in liquid nitrogen. In this step, internal standards were also added (10 ng/µL d_4_SA; 4 ng/µL d_5_JA; 2 ng/µL d_6_ABA). The mixture was shaken overnight (at least 18 h; 200 rpm; 4 °C). After incubation, the salt mixture (magnesium sulfate anhydrous, sodium chloride) was added, and the mixture was then vigorously mixed for 1 min. The mixed samples were centrifuged at 10,000× *g* for 10 min to obtain the supernatant. The clean-up of the supernatant was performed by vigorously mixing the supernatant with sodium sulfate for 5 min, followed by centrifugation (10,000× *g* for 10 min). The supernatant was collected and dried using nitrogen gas. Samples were dissolved using 1 mL 1 M FA and subjected to solid phase extraction (SPE) using silica packed columns (Discovery^®^ DSC-18 SPE Tube; Supleco). The columns were activated with 100% methanol and conditioned with 1 M FA. The applied samples were purified twice using 1 M FA. Elution was performed using 80% methanol (*v*/*v*). Each sample was lyophilized, suspended in 100 µl 35% methanol (*v*/*v*), and centrifuged. For phytohormone determination, LCMS-8045 tandem mass spectrometry (Shimadzu Corporation, Kyoto, Japan) was used. Chromatographic separation was performed on a Kinetex^®^ 2.6 μm XB-C18 100 Å reversed-phase column (150 mm × 2.1 mm). Water with 0.1% formic acid (*v*/*v*) (A) and methanol with 0.1% formic acid (*v*/*v*) (B) were used as the mobile phase. Separation was performed on a linear gradient of 40–90% (*v*/*v*) methanol for 7 min at a flow rate of 0.3 mL/min at 30 °C. In mass spectrometry, the samples were subjected to negative electrospray ionization (ESI) and ions were fragmented by collision-induced dissociation (CID). The ionization voltage was −3 kV. Analysis of individual phytohormones was based on multiple reaction monitoring (MRM).

### 3.5. Determination of Endogenous Levels of Cyclic Nucleotides

Endogenous levels of cyclic nucleotides were measured using mass spectrometry combined with liquid chromatography (LC–MS). Cyclic nucleotides were extracted using a previously described method [54] for 100 mg of tissue.

### 3.6. Gene Expression Analysis

Total RNA was isolated from plant tissue using a GeneMATRIX Universal RNA Purification Kit (EURx) and digested using DNase I (Thermo Scientific) according to the manufacturer’s instructions. First-strand cDNA was synthesized from 1 μg of total RNA using random hexamers and GoScript™ Reverse Transcriptase (Promega) following the manufacturer’s instructions. The gene-specific primers and hydrolysis probes for qPCR were designed with ProbeFinder software from the Universal Probe Library Assay Design Center (Roche) and Primer3Plus software. The β-actin gene was used as a reference. To determine the PCR efficiencies, standard curves for both target and control genes were obtained using a series of cDNA dilutions as a template. The RT–qPCR was performed on a LightCycler^®^ 480 using LightCycler 480 Probes Master following the manufacturer’s protocol (Roche). Three independent biological replicates and three technical replicates were analyzed. Relative levels of gene expression were calculated according to the Pfaffl method [55]. A list of the PCR primers and probes used for the experiments is provided in Table S1.

### 3.7. Data Analysis

The statistical significance of differences between the mean values of the experimental variants of the analyzed parameters was determined using two-way ANOVA followed by Tukey’s test in SigmaPlot 14.5 (Systat Software). Differences of *p* < 0.05 were considered significant. The mean and standard deviation were calculated. Error bars shown in all figures represent the standard deviation calculated from three repetitions of each experiment.

## 4. Conclusions

Under natural conditions, plants are exposed to attacks from a range of pathogens and pests that possess a variety of infection strategies. Cross-talk between various elements allows the plant to divert resources to the most appropriate defense mechanisms. Much attention is currently focused on unraveling the molecular mechanisms involved in plant signal transduction cascades during the infection process. The steps between pathogen perception and the initiation of cellular defense response programs in plants remain only partially understood. The work presented herein supplements this knowledge with respect to cyclic nucleotides and purine nucleotide cyclases. The observations made here allow us to conclude that cGMP is an element involved in the processes triggered as a result of infection and changes in its levels affect stress hormones, especially jasmonic acid. Therefore, stimuli-induced transient elevation of cGMP in plants may play beneficial roles in priming an optimized response, likely by triggering the mechanisms of feedback control.

## Data Availability

Data are contained within the article or Supplementary Material.

## Supplementary Materials

The following supporting information can be downloaded at: https://www.mdpi.com/article/10.3390/ijms23052674/s1.
