# Supplementary material for: BdGUCD1 and Cyclic GMP Are Required for Responses of Brachypodium distachyon to Fusarium pseudograminearum in the Mechanism Involving Jasmonate
Source: Int J Mol Sci. 2022 Feb 28;23(5):2674. doi: 10.3390/ijms23052674 (PMC8910563; doi:10.3390/ijms23052674)
Supplement: Supplementary file 1 [file ijms-23-02674-s001.zip › ijms-1602899-supplementary.pdf]

Supplementary Methods

Table S1. Sequence of the primers employed for PCR amplification in this study. The table includes oligonucleotide sequences employed for cloning (lower case letters indicate the SalI and NotI restriction sites, respectively) and RT-qPCR.

	**gene symbol**	**primers sequence 5'->3'**	
1	*Bd*GUCD1 (for cloning)	F	ATTCCCGGgtcgacTATGGTGCTCAGGACGCTAG
		R	AGTCACGATgcggccgcTTACAGAGAGCTGTCTGGAAGTTTGC
2	*Bd*PepR2 (for RT-qPCR)	F	CGTGTACAGCTACGGAGTCG
		R	GCAGAATCCACAGGCATCTT
probe #128 (cat. no. 04693647001)			
3	*Bd*GUCD1 (for RT-qPCR)	F	GACATCGATAGAGTGGACGAACT
		R	GCACTGATGGATCTGCATTG
probe #151 (cat. no. 04694376001)			
4	*Bd*ACT1 (for RT-qPCR)	F	TGCTCCTCCTGAAAGGAAGT
		R	ACTCAGCCTTTGCAATCCAC
probe #120 (cat. no. 04693540001)			

Table S2. The qPCR efficiency values for the genes analyzed.

	**Gene**	**Efficiency**
1	***Bd*PepR2**	1,983
2	***Bd*GUCD1**	1,977
3	***Bd*ACT1**	2,000

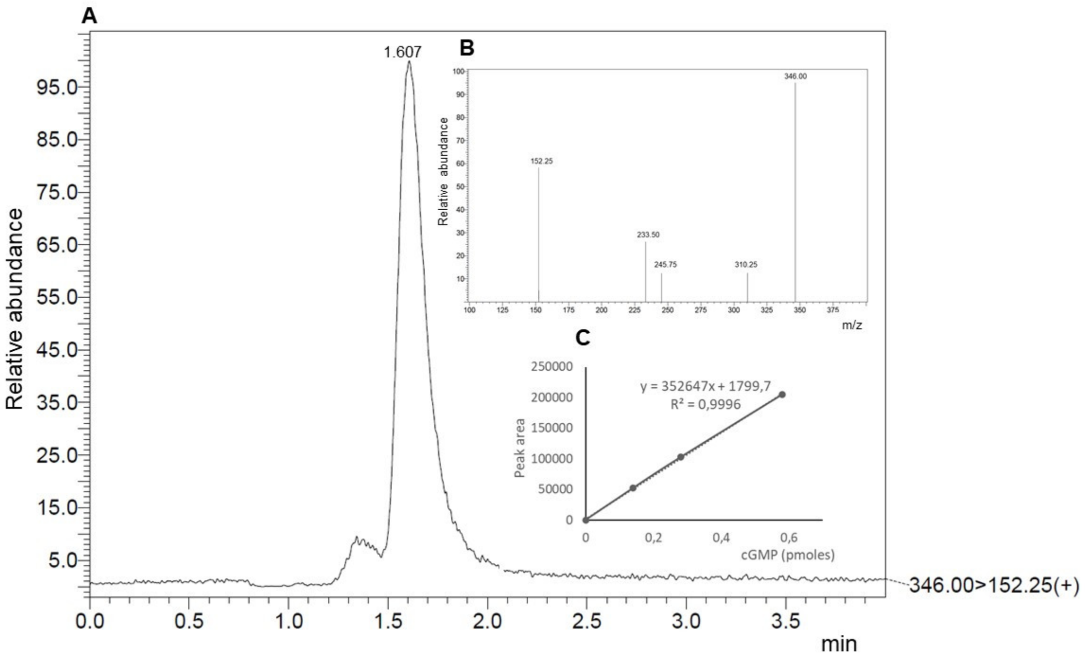

Figure S1. Detection of cGMP generated by BdGUCD1 by LC-MS/MS. (A) Determination of GC level by LC-MS/MS. Ion chromatogram of cGMP was generated from a reaction mixture containing 5 µg of purified protein (B) Inset showing parent cGMP ion at m/z 346.00 [M + H]+ and corresponding fragmented daughter ion at m/z 125.25 [M + H]+. Fragmented product ion was used for quantitation. (C) Inset showing the cGMP calibration curve performed with 0–0.58 pmoles of pure cGMP on the column.

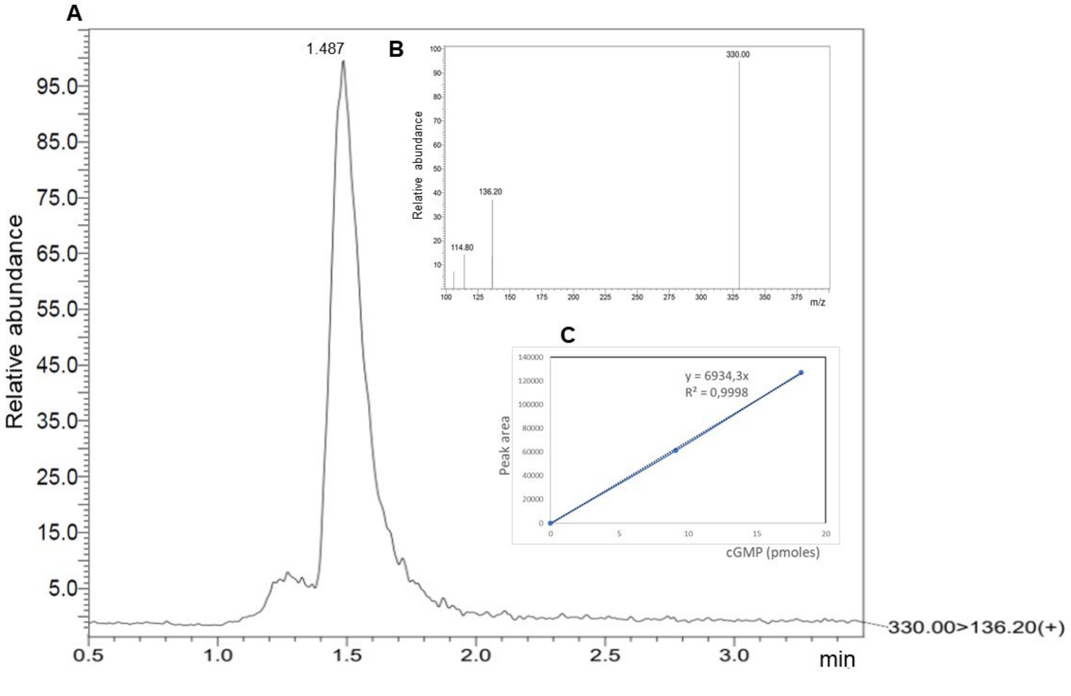

Figure S2. Detection of cAMP generated by BdGUCD1 by LC-MS/MS. (A) Determination of AC level by LC-MS/MS. Ion chromatogram of cAMP was generated from a reaction mixture containing 5 µg of purified protein or (B) Inset showing parent cAMP ion at m/z 330.00 [M + H]+ and corresponding fragmented daughter ion at m/z 136.20 [M + H]+. Fragmented product ion was used for quantitation. (C) Inset showing the cGMP calibration curve performed with 0–18.22 pmoles of pure cAMP on the column.
